# Acoustic Radiation Force Impulse (ARFI) and Transient Elastography (TE) for evaluation of liver fibrosis in HIV-HCV co-infected patients

**DOI:** 10.1186/1471-2334-14-405

**Published:** 2014-07-21

**Authors:** Nora Frulio, Hervé Trillaud, Paul Perez, Julien Asselineau, Marianne Vandenhende, Mojgan Hessamfar, Fabrice Bonnet, Florent Maire, Jean Delaune, Victor De Ledinghen, Philippe Morlat

**Affiliations:** 1 Department of Diagnostic and Interventional Imaging, Saint-André hospital, CHU de Bordeaux, 1 rue Jean Burguet, 33075 Bordeaux, France; 2 Unité de Soutien Méthodologique à la Recherche Clinique (USMR), CHU Bordeaux Pôle de Santé Publique, place Amélie Rabat-Leon, 33000 Bordeaux, France; 3 Department of internal medicine and infectious diseases, Saint-André hospital, CHU de Bordeaux, 1 rue Jean Burguet, 33075 Bordeaux, France; 4 INSERM U1053, Bordeaux university, Bordeaux, France; 5 Centre d’investigation de la fibrose hépatique, Haut-Lévêque hospital, CHU de Bordeaux, 1 Avenue de Magellan, 33604 Pessac, France

**Keywords:** ARFI elastography, Liver fibrosis, HIV-HCV co-infected patients, Transient elastography

## Abstract

**Background:**

Transient elastography (TE) is widely used for non-invasive assessment of liver fibrosis in HIV-HCV co-infected patients. TE, however, cannot determine liver morphology. Acoustic radiation force impulse (ARFI) imaging is a novel procedure enabling assessment of liver fibrosis during a conventional ultrasonographic examination. This study evaluated the correlation between liver fibrosis measurements by TE and ARFI.

**Methods:**

Each of 46 HIV-HCV patients underwent both ARFI and TE within 6 months. Patients were evaluated by the “equivalent METAVIR” scoring system, using previously established cut-off values. Agreements between the ARFI and TE scores were estimated by Kappa coefficients, with Kappa values ≥0.40, ≥0.60, and ≥0.80 defined as moderate, good and very good agreement, respectively.

**Results:**

ARFI and TE yielded "Equivalent Metavir" fibrosis scores of F1 in 26 and 31 patients, respectively; F2 in nine and seven, respectively; F3 in three and two, respectively; and F4 in eight and six, respectively. The two methods showed very good agreement in predicting overall stages [Kappa = 0.82] and for F ≥3 [Kappa = 0.80] and moderate agreement in predicting significant fibrosis F ≥2 [Kappa = 0.50]. Morphologic ultrasound analysis concomitant to ARFI detected two hepatocarcinomas.

**Conclusions:**

ARFI showed promising results in the non-invasive assessment of liver fibrosis in HIV-HCV patients, with liver fibrosis staging similar to that of TE. Moreover, ARFI can assess morphology and fibrosis during the same session.

## Background

Since the introduction of highly active antiretroviral therapy (HAART), chronic liver disease has emerged as one of the leading causes of morbidity and mortality in HIV-HCV co-infected patients [1,2]. Therefore, the management of HCV-related liver disease has become a major challenge in these patients [3].

Evaluation of liver fibrosis is considered essential to the management of patients with chronic hepatitis C, providing prognostic information and assisting in making therapeutic decisions [4-7]. To date, liver biopsy has been considered the gold standard to assess activity and fibrosis in patients with chronic liver disease. Despite its diagnostic utility, liver biopsy is limited because of its invasiveness and cost, the risk of complications including death, its poor acceptance by patients, the lack of availability of expert practitioners, and intra/inter-observer variability [8-10]. In recent years, various non-invasive methods for liver fibrosis assessment, have been developed, including serum biomarkers and elastography techniques. Transient elastography (TE), real-time elastography (RTE), acoustic radiation force impulse imaging (ARFI), and shear wave elastography are the most frequent elastography techniques [11-15].

TE (FibroScan®) is currently the most extensively used elastography technique in clinical practice [16-19]. Many prospective studies have evaluated the diagnostic performance of TE in staging liver fibrosis in patients with chronic liver diseases, including chronic viral hepatitis C [11,20-22], chronic viral hepatitis B [23], HIV–HCV co-infection [24-26], alcoholic disease [27], and non-alcoholic fatty liver disease [28]. Two meta-analyses found that TE is highly accurate for the diagnosis of cirrhosis and advanced fibrosis [16,17], suggesting that TE may be used in place of biopsy in most patients with chronic hepatitis C [18,29].

TE, however, has several drawbacks, including the requirement for specific equipment that performs only elastography, its ability to assess only the right liver, and its inability to visually determine the site of measurement. Moreover, ascites and obesity are limiting factors. ARFI imaging has many advantages over TE. The technology used for ARFI has been incorporated into a conventional ultrasound system, allowing ultrasound analysis of liver morphology at the same time. In addition, ARFI allows elastography to be performed with a flexible metering box at variable depths, enabling the exact localization of the measurement site, and the examination can also be performed in patients with ascites. ARFI measurements have been found to correlate well with the stage of hepatic fibrosis [30-36]. However, to our knowledge, no study to date has investigated the potential benefits of ARFI elastography in evaluating fibrosis stage in HIV-HCV co-infected patients. This study therefore assessed the agreement between TE and ARFI for the evaluation of liver fibrosis in this patient population.

## Methods

### Patients

The study was approved by the institutional review board of our University hospital. Between July 2009 and May 2012, HIV-HCV co-infected patients regularly followed up at the HIV out-patient Clinic of the Department of Internal Medicine and Infectious Disease of our hospital were prospectively enrolled. All patients provided written informed consent before inclusion.

The demographic, clinical and biological information of all patients was obtained from their medical records. Factors included patient age, body mass index (BMI), alcohol consumption, medications, specific antiretroviral drugs, plasma HIV-RNA levels (IU/ml), complete blood counts, absolute number of CD4+ T-cells (/mm^3^), plasma HCV- RNA concentrations (IU/ml), and HCV genotype. Men and women were considered heavy drinkers if they consumed >3 and >2 glasses of alcohol per day, respectively. Alanine aminotransferase (ALT), aspartate aminotransferase (AST), γ-glutamyl transferase (GGT), alkaline phosphatase (ALP), and bilirubin concentrations were also measured.

The presence of cirrhosis was evaluated in all patients histologically and from imaging, clinical and biological results [37,38]. Imaging findings indicative of cirrhosis included small nodular and irregular livers with increased echogenicity and/or a significant reduction in Doppler flow in the portal circulation on ultrasound, CT, or MRI.

Quantitative ARFI imaging (Acuson S2000 Siemens) and TE were performed by two different observers, with each subject undergoing the two examinations no more than 6 months apart. The physician performing the second procedure was blinded to the results of the first procedure.

### Transient elastography (TE)

TE was performed in the right lobe of the liver through the intercostal space as described [39,40]. Liver stiffness was measured in a volume approximating a cylinder 1 cm wide and 4 cm long, and at a depth between 25 and 65 mm. As suggested by the manufacturer, 10 successful acquisitions were obtained for each patient in fasting conditions. TE results were defined as very reliable (IQR/M ≤0.10), reliable (0.10 < IQR/M ≤0.30 or IQR/M >0.30 with LSE median <7.1 kPa) and poorly reliable (IQR/M >0.30 with LSE median ≥7.1 kPa) [41]. Poorly reliable results were excluded. The median value for each patient was expressed according to “equivalent Metavir” fibrosis scores using previously determined cut-off values for HCV mono-infected patients of 7.1, 9.5 and 12.5 kPa for liver fibrosis scores of F ≥ 2, F ≥3, and F = 4, respectively [11].

### ARFI examination

**Figure 1:**
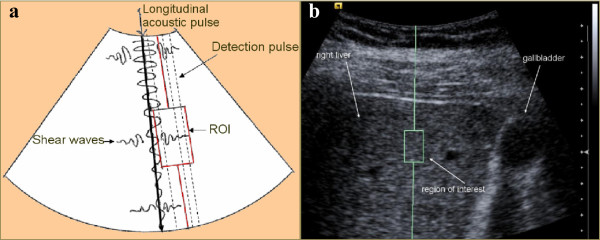
**Principle or ARFI quantitative measurement. a)** Schematic of principles of ARFI imaging in virtual touch quantification mode – **b)** Example of a SWV measurement of the right liver. Transmission of a longitudinal acoustic pulse leads to tissue displacement, resulting in shear wave propagation away from the region of excitation. The shear wave velocity is measured within a defined region of interest (ROI) using ultrasound tracking beams laterally adjacent to the single push beam. The shear velocity is estimated in a graphically displayed ROI measuring 1 × 0.5 cm. The shear wave propagation velocity is proportional to the square root of tissue elasticity.

ARFI imaging is a method of quantifying the mechanical properties of tissues, without manual compression, by measuring the shear wave velocity induced by acoustic radiation and propagating in the tissue. This quantitative technique provides a single unidimensional measurement of tissue elasticity like TE, although the measurement area can be positioned on a two-dimensional B mode image. The region is a 1 × 0.5-cm rectangle, which can be freely moved in the two-dimensional B mode image to a maximum depth of 8 cm from the skin plane. Measurements are expressed as m/s, indicating shear wave speed traveling perpendicular to the shear wave source. The principle of ARFI is described in Figure 1[30,42,43].

All patients underwent quantitative elastographic examinations in fasting conditions with a Siemens Acuson S2000TM ultrasound system (Siemens AG, Erlangen, Germany) and an abdominal curved transducer (4C1), using the virtual touch tissue quantification system.

The first step assessed liver morphology and echo-structure, looking for cirrhosis, signs of portal hypertension and tumor lesions. In the second step, 10 ARFI measurements of the right liver through the intercostal space were performed by a single examination. To standardize these examinations, an area of liver tissue free of large blood vessels was chosen at a minimal distance of 2 cm below the liver capsule. ARFI measurements were expressed in m/s as median (M), mean and standard deviation, and interquartile range (IQR), with the IQR/M ratio used to evaluate the variability of the measurements. An IQR/M < 0.3 was considered a homogeneous set of measurements. Median ARFI value was expressed according to the ‘equivalent Metavir’ score using previously determined cutt-off values for HCV mono-infected patients of 1.34, 1.55, 1.80 m/s for liver fibrosis scores of F ≥ 2, F ≥3, and F = 4, respectively [44].

### Statistical analysis

Quantitative characteristics were expressed as mean (standard deviation), median (first-third quartile) and range, and compared using non-parametric Wilcoxon statistics. Qualitative characteristics were expressed as frequencies and proportions, and compared using Fisher’s exact test.

Correlations between TE and ARFI measurements were estimated using Spearman’s correlation coefficient and its two-sided 95% confidence interval (CI), as calculated with Fisher's z transformation.

Agreements between “equivalent Metavir” fibrosis stages on ARFI and TE were assessed by simple Kappa coefficients for predictions of F ≥ 3 or F ≥2, and F = 4, and weighted Kappa coefficients for predictions of F1 to F4 Metavir scores; two-sided 95% CIs were also estimated. Kappa values ≥0.60 and ≥0.80 were considered indicative of good and very good agreement, respectively. Additional analysis was performed in patients infected with HCV genotypes 1 and 4, who usually have a poorer response than patients infected with genotypes 2 and 3.

Quantitative and qualitative variables associated with discordances between ARFI and TE staging were assessed by the Wilcoxon test and Fisher’s exact test, respectively. Only univariate analyses were performed because of the small number of subjects.

## Results

### Characteristics of the study population

Of the 86 examinations, 40 were excluded because of missing data (n = 8), too long delay (>6 months) between ARFI and TE (n = 24) or repeated examinations for the same patient (n = 8). When liver stiffness was measured repeatedly in the same patient, only the measurement with minimal delay between ARFI and TE was included in the analysis. Valid results of both ARFI and TE were obtained for 46 patients; their demographic and clinical and biological characteristics are summarized in Table 1.

**Table 1:** Patient demographic and clinical characteristics

**Demographics**	
Age (y) Median (Q1; Q3)	48 (45; 51)
Gender n (%)	
Female	14 (30.4)
Male	32 (69.6)
BMI Median (Q1; Q3)	22.4 (19.8; 24.3)
Overweight (BMI > =25) n (%)	8 (17.4)
Chronic alcohol abuse n (%)	15 (34.1)
Cirrhosis n (%)	6 (13)
Child Pugh stage n	6
A n (%)	6 (100)
B, C, D n	0
**HIV treatment**	
Protease inhibitors n (%)	28 (60.9)
Integrase inhibitors n (%)	3 (6.5)
Non-nucleoside RT inhibitors n (%)	8 (17.4)
Other treatment n (%)	7 (15.2)
**HCV treatment**	
None, n (%)	28 (60.9)
Previous treatment failure n (%)	10 (21.7)
Previous sustained virologic response n (%)	8 (17.4)
**Virology**	
HCV genotype n (%)	
1-1a-1b	24 (59)
2-3-3a	9 (22)
4 41b-4a	8 (19)
ND	5 (10)
HCV RNA > 15 IU/ml n (%)	38 (83)
HCV RNA IU/ml (Q1; Q3)	1116144 (391800; 3680000)
Undetectable HIV RNA n (%)	40 (87)
CD4/mm3 (Q1; Q3)	576 (377; 833)
**Liver blood test**	
Delay between ARFI and blood test days (Q1; Q3)	0 (0; 1)
AST or ALT ≥2N n (%)	7 (15.6)
GGT and/or ALP ≥2N n (%)	15 (33.3)
Bilirubin ≥2N n (%)	10 (22.2)
**US liver characteristics**	
Steatosis n (%)	3 (6.5)
Cirrhosis in US n (%)	6 (13)
US portal hypertension signs n (%)	2 (4.3)
US hepatomegaly n (%)	14 (30.4)
US focal liver lesion n (%)	5 (10.9)
**ARFI characteristics**	
Median (Q1; Q3) (m/s)	1.29 (1.15; 1.55)
Min; Max	0.93; 2.86
**TE characteristics**	
Median (Q1; Q3) (kPa)	6.1 (4.6; 7.2)
Min; Max	3.4; 35.3

Of the 46 patients, 28 did not receive any treatment for HCV, whereas the other 18 were treated with peginterferon and ribavirin (SOC). At the time of ARFI, eight had achieved a sustained virologic response, whereas the other 10 had failed treatment. No patient received any direct-acting antiviral agents (DAAs). Moreover, all 46 patients had received highly active antiretroviral therapy (HAART), including 28 who received third-generation protease inhibitors (PIs), three who received integrase inhibitors (INIs), eight who received non-nucleoside reverse transcriptase inhibitors (NNRTIs), and seven who received other treatment combinations (Table 1).

Ultrasound analysis allowed the detection of focal liver lesions in five patients, including two with hepatocellular carcinoma (HCC) and three with benign liver lesions. The two HCCs presented with arterial hypervascularity and wash-out in venous and delayed phase on dynamic enhanced MRI [45]. Their diagnosis was confirmed by histologic examination of biopsy specimens obtained just before liver nodule radiofrequency. The three benign liver lesions were diagnosed based on their tumor-specific vascularity patterns in the arterial, portal, and late phases on dynamic enhanced MRI, as described [46-48]. Imaging features were typical, with liver biopsies not required to confirm their diagnosis.

### Evaluation of liver fibrosis

**Figure 2:**
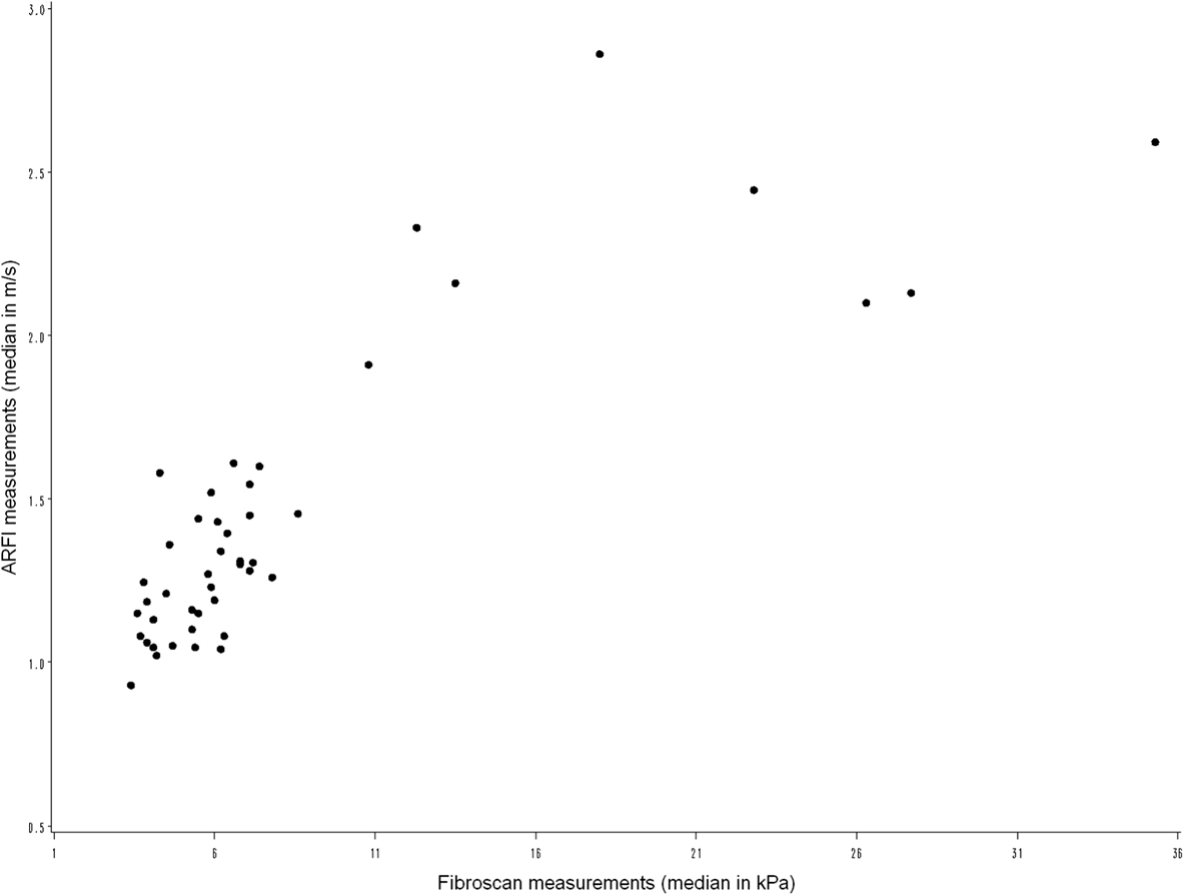
Scatterplot of the relationship between median ARFI (m/s) and TE (kPa) measurements.

The median delay between ARFI and TE in the 46 evaluated patients was 10 days (range, 0–25 days). Median liver stiffness on TE was 6.1 kPa (range 3.4–35.3 kPa) and median shear wave velocity (SWV) on ARFI was 1.29 m/s (range 0.93–2.86 m/s). Median liver stiffnesses on TE in patients with F1, F2, F3, and F4 fibrosis stages were 5.4 kPa (range 4.1–6.1 kPa), 7.2 kPa (range, 7.1–7.8 kPa), 11.6 kPa (range, 10.8–12.3 kPa) and 24.6 kPa (range, 18.0–27.7 kPa), respectively. Median SWVs on ARFI in patients with F1, F2, F3 and F4 fibrosis stages were 1.15 m/s (range, 1.06–1.25 m/s), 1.44 m/s (range, 1.40–1.47 m/s), 1.60 m/s (range, 1.58–1.61 m/s), and 2.25 m/s (range, 2.12–2.52 m/s), respectively. Figure 2 shows a scatterplot of the relationship between median results on TE and ARFI.

### Comparison of equivalent Metavir fibrosis scores on TE and ARFI

Spearman correlation analysis showed that the correlation coefficient between ARFI and TE measurements was 0.76 [95% CI, 0.61–0.86]. Table 2 shows the agreement between TE and ARFI measurements, as determined by equivalent Metavir fibrosis scores. Overall agreement between the two methods was very good [concordance 69.6%, weighted Kappa = 0.82; 95% CI = 0.70–0.95]. Agreement was also very good for predicting severe fibrosis (≥F3) [concordance 93.5%, Kappa = 0.80, 95% CI = 0.59–1.00], and was moderate for predicting significant fibrosis (≥F2) [concordance 76.1%, Kappa = 0.5, 95% CI = 0.25–0.75].

**Table 2:** Agreement between TE and ARFI fibrosis stage classification according to “equivalent Metavir” fibrosis scores

		**ARFI**				
		**F1**	**F2**	**F3**	**F4**	**TOTAL**
**TE**	**F1**	23	6	2	0	31
	**F2**	3	3	1	0	7
	**F3**	0	0	0	2	2
	**F4**	0	0	0	6	6
	**TOTAL**	26	9	3	8	46

Observed agreement: 69.6% 95% CI = [54.2–82.3].

Weighted Kappa coefficient: 0.82 95% CI = [0.70–0.95].

Of the 46 patients, 14 (30%) showed discordant Metavir scores between the two methods, 11 in predicting F ≥ 2 and three in predicting F ≥ 3. ARFI yielded a higher fibrosis score than TE for 11 of these patients. As the discordance was more important for predicting F ≥ 2, factors possibly associated with these discordances were analyzed, including age; sex; BMI; alcohol consumption; plasma HCV-RNA levels; absolute CD4+ (/mm^3^) cells; HCV genotype; increased serum AST, ALT, ALP, and GGT (>2N) and bilirubin concentrations; steatosis; cirrhosis; HIV and HCV treatment; and response to HCV treatment. Only age was associated with the discordance between the two methods (p = 0.047) with older patients in the discordant group. Discordance also tended to be associated directly with hepatomegaly and inversely with overweight, cytolysis, and cholestasis. No significant difference was found with HIV (p=0.22) and HCV(p=0.55) treatment.

Similar results were observed in the subgroup of patients infected with HCV genotypes 1 and 4. Table 3 shows the agreement between TE and ARFI measurements, as determined by equivalent Metavir fibrosis stage classification, in these patients. Agreement between the two methods was very good overall [concordance 65.6%, weighted Kappa = 0.84; 95% CI = 0.71–0.96], very good in predicting severe (F ≥3) fibrosis [concordance 93.8%, Kappa = 0.83, 95% CI = 0.61–1.00], and moderate in predicting significant fibrosis (F ≥2) fibrosis [concordance 75%, Kappa = 0.51, 95% CI = 0.22–0.79].

**Table 3:** Agreement between TE and ARFI fibrosis stage classification according to “equivalent Metavir” fibrosis stage in patients infected with HCV genotypes 1 and 4

		**ARFI**				
		**F1**	**F2**	**F3**	**F4**	**TOTAL**
**TE**	**F1**	13	5	1	0	19
	**F2**	2	3	1	0	6
	**F3**	0	0	0	2	2
	**F4**	0	0	0	5	5
	**TOTAL**	15	8	2	7	32

Overall proportion of agreement: 65.6% 95% CI [46.8–81.4].

Kappa: 0.84 95% CI [0.71–0.96].

## Discussion

The correct evaluation of liver fibrosis in patients with chronic liver disease, especially in those co-infected with HIV and HCV, is of paramount importance for patient management. The presence of significant fibrosis is an indication for treatment, regardless of HCV genotype, whereas the presence of cirrhosis is an indication for initiating a surveillance program for the early detection of HCC and for esophageal varices.

To our knowledge, no study to date has used ARFI to evaluate liver fibrosis in HIV-HCV co-infected patients or compared the results of ARFI and TE in this population. This pilot study, found that these two methods yielded similar results. ARFI and TE showed very good agreement in predicting overall fibrosis stage and stage F ≥ 3. According to the literature, the two methods also showed similar results in mono-infected patients and in patients with other types of chronic liver disease. A meta-analysis of nine studies showed that ARFI imaging had excellent diagnostic accuracy for the staging of liver fibrosis in various chronic liver diseases; compared with liver biopsy, ARFI was highly accurate in the diagnosis of fibrosis stage F ≥ 3 (AUROC = 0.91) and for the diagnosis of liver cirrhosis (AUROC = 0.93) [44]. Moreover, an international multicenter study of 911 HCV mono-infected patients found that ARFI was highly accurate in the diagnosis of fibrosis stage F ≥ 3 (AUROC = 0.83) [49].

We observed a moderate agreement between the two techniques for predicting F ≥ 2. Earlier studies also showed that TE or ARFI was more accurate for predicting extreme stages (F1 or F4) than moderate (F2) fibrosis. For example, the AUROCs for ARFI were higher for predicting F=4 (0.84;0.95;0.91) than F≥ 2 (0.79;0.77;0.82) [30,33,49].

In addition, agreement between the TE and ARFI scores was determined in patients infected by genotype 1 and 4 who usually have poorer response than patients infected with genotype 2 and 3. Similarly, there was no difference in concordance level between patients with genotypes 1 and 4 and those with other genotypes. The TE and ARFI methods showed very good agreement in predicting overall fibrosis stage and F ≥ 3 in patients with genotypes 1 and 4.

This study included a small number of patients (n = 46). However, it was designed as a pilot study, with intraclass correlation coefficients precise enough to draw conclusions by comparing the lower limit of the 95% CIs with 0.6, deemed to represent the lower limit of good agreement.

ARFI has several advantages compared with TE. TE has been reported to have an average failure rate of 3.1% and to be highly dependent on BMI. Moreover, TE measurements were estimated to be unreliable in 15.8% of patients in a very large study [19]. We found that ARFI examinations were reliable in all patients, with no measurement limitations due to overweight or the presence of ascites. ARFI measurement depth can be adapted according to the distance between the skin and liver capsule. In addition, ARFI is an ultrasound-based elastography method, integrated into a routine ultrasound machine, allowing simultaneous morphological and Doppler analysis of the liver, screening for focal liver lesions and evaluation of liver fibrosis. Moreover, the presence of B mode allows ARFI measurements to be localized to liver segments far from the capsule and blood vessels. Ultrasound morphological analysis of our patients identified cirrhosis in six patients and focal liver lesions in five, including two HCCs. Moreover, the presence of HCC did not influence fibrosis evaluation because ARFI measurements in non-tumoral liver were performed at a distance (>3 cm) from the tumor. Indeed, the visual control in B mode allowed the ROI to be positioned to avoid specific areas, including tumors.

TE and ARFI showed a discrepancy in predicting fibrosis score in 30% of patients, especially in predicting F ≥ 2. Apart from age, no factor was clearly associated with these discrepancies. However, the study size was not powered for this aim. Liver biopsy should have been performed in patients with discordance, but was not except in one patient, in whom ARFI, but not TE, agreed with biopsy results. Moreover, when a discrepancy was observed between the two techniques, it was not possible to conclude whether TE or ARFI was more correct. These discrepancies may be linked to the cut-off values used, as these are not the same in all published studies. The TE cut-off values we used [11] yielded similar results as other cut-off values from a recent meta-analysis [17] (data not shown). However, although meta-analyses have found TE to be reliable in assessing liver fibrosis stage, irrespective of etiology, most studies were performed in western patients with HCV mono-infection. Thus, applying these cut-off values to patients co-infected with HIV and HCV may result in discrepancies. To date, however, no ARFI cut-off values have been determined for co-infected patients.

Finally, although liver biopsy has been considered the gold standard for the assessment of liver fibrosis, we elected to use non-invasive methods for comparison [12]. Fibrosis is heterogeneously distributed throughout the liver, whereas a biopsy evaluates only 1/50000 of the total volume of the liver. The likelihood of misdiagnosing a fragment 2.5 cm in length has been estimated to be as high as 25%, with the optimal size of a biopsy sample being 4 cm, which is difficult to obtain in daily practice [10]. Moreover, liver biopsy is an invasive and costly method, with high risks of complications and intra-/inter-observer variability. Thus, liver biopsy is increasingly replaced by non-invasive methods, especially TE. Since TE is considered a reliable diagnostic modality in clinical practice, we compared the results of ARFI with those of TE.

## Conclusions

This pilot study showed good agreement between TE and ARFI in evaluating liver fibrosis in HIV-HCV co-infected patients, suggesting that ARFI may replace TE in this population. In addition to being a new and promising sonography-based method for non-invasive assessment of liver fibrosis, ARFI is cost effective, allowing routine ultrasound examination and fibrosis assessment during the same session.

### Consent and ethics committee

Written informed consent was obtained from all patients for publication of this paper; copies are available for review by the Editor of this journal.

This study and its publication were approved by our local ethics committee: CPP Sud-Ouest et Outre Mer III Bordeaux.

## Abbreviations

AASLD: American Association for the Study of Liver Diseases; ALP: Alkaline phosphatase; ALT: Alanine aminotransferase; ARFI: Acoustic radiation force impulse; AST: Aspartate aminotransferase; AUROC: Area under the receiver operating characteristic curve; BMI: Body mass index; CT: Computed tomography; DAA: Direct-acting antiviral agents; GGT: γ-glutamyl transferase; HAART: Highly active antiretroviral therapy; HCC: Hepatocellular carcinoma; HCV: Hepatitis C virus; HIV: Human immunodeficiency virus; INIs: Integrase inhibitors; IQR: Interquartile range; LB: Liver biopsy; LSE: Liver stiffness elastography; MRI: Magnetic resonance imaging; ND: Not done; NNRTIs: Non-nucleoside reverse transcriptase inhibitors; PIs: Protease inhibitors; RNA: Ribonucleic acid; ROI: Region of interest; RT: Reverse transcriptase; RTE: Real-time elastography; SOC: Standard of care; SWE: Shear wave elastography; SWV: Shear wave velocity; TE: Transient elastography; US: Ultrasound.

## Competing interests

The authors state that they have no competing interests.

## Authors’ contributions

NF, HT, and PM designed and supervised the study. NF supervised all ARFI measurements. NF, JD, and FM, extracted the data. PP and JA analyzed the data. NF, PM, PP, and JA interpreted the data. NF wrote the manuscript. PM, JA, PP, and HT drafted and finalized the manuscript. All authors revised the manuscript critically for important intellectual content. All authors read and approved the final manuscript. All authors are permanent employees of the Bordeaux University Hospital, which funded this study and the preparation of this manuscript.

## Pre-publication history

The pre-publication history for this paper can be accessed here:

http://www.biomedcentral.com/1471-2334/14/405/prepub
